# Radix Actinidia chinensis Suppresses Renal Cell Carcinoma Progression: Network Pharmacology Prediction and In Vivo Experimental Validation

**DOI:** 10.1155/2022/3584445

**Published:** 2022-07-30

**Authors:** Biao Liu, Liang Zhang

**Affiliations:** College of Pharmaceutical Science, Zhejiang University of Technology, Hangzhou, China

## Abstract

**Background:**

Renal cell carcinoma (RCC) is a frequent disease with limited curative methods. This study is aimed at investigating the role and mechanism of Radix Actinidia chinensis (RAC) on RCC.

**Methods:**

The ingredients, target, and crucial pathways of RAC in RCC therapy were analyzed by network pharmacology. Then, an RCC animal model was established by subcutaneously injecting A498 cell suspension to BALB/c nude mice. After 1 week, the mice in the RAC-L/M/H groups were administered with RAC at 5, 10, and 20 mg/kg/d, respectively. The histopathology of the tumor was evaluated. The contents of tumor inflammatory cytokines and serum oxidative stress factors were detected by ELISA. The apoptosis of tumor tissues was assessed by TUNEL staining. The expressions of apoptosis-, proliferate-, autophagy-, and MAPK-related proteins were measured.

**Results:**

There were 13 active ingredients, and 20 RCC-relevant targets were selected from RAC; KEGG pathway indicated that these targets were enriched in the PI3K/AKT/mTOR and MAPK pathway. In *in vivo* experiments, RAC not only obviously damaged tumor cells and decreased the release of inflammatory cytokines and oxidative stress factors but also enhanced the apoptosis of the tumor cell in RCC mice. Besides, the expressions of apoptosis-, proliferate-, autophagy-, PI3K/AKT/mTOR path-, and MAPK path-related proteins were all affected by RAC.

**Conclusion:**

RAC attenuated RCC by regulating inflammation response, oxidative stress, apoptosis, proliferation, and autophagy, and its effects were partly linked to the PI3K/AKT/mTOR and MAPK pathway, which indicated that RAC may be a candidate drug for RCC.

## 1. Introduction

Renal cancer is a prevalent malignant tumor of the urinary system, and renal cell carcinoma (RCC) is a common pathological type of renal cancer (about 90%) [1]. During the past 10 years, the incidence of RCC worldwide has increased annually, and nearly 30% of the patients are found to have metastatic lesions at the time of initial diagnosis [2]. Although surgery remains the first choice for treating RCC, its overall prognosis is still poor [3]. At present, the treatment options for RCC patients are very limited besides surgery [4]. It is reported that targeted biologics, such as tyrosine kinase inhibitor [5] and rapamycin inhibitor [6], have exhibited certain clinical efficacy for RCC. Nevertheless, these mentioned biologics easily produce drug resistance and negative adverse effects [7]. Therefore, it is urgent to search for more effective and safer drugs for the treatment of RCC.

Inflammation response and oxidative stress are believed as important risk factors that trigger and promote RCC, and they interact with each other [8]. Specifically, inflammation response may promote the development of cancer by enhancing the proliferation, metastasis, and invasion of cancer cells, aiding cancer cells to escape from immune surveillance, as well as inducing resistance of drug [9]. Oxidative stress can induce the oncogenesis and accelerate the progression of tumor via breaking DNA and activating cellular signal transduction pathways, which are linked to the malignant transformation [10]. As generally known, cancer cells often display aberrant apoptosis, proliferation, and autophagy to facilitate the growth and survival of tumor [11]. Thus, the modulation of abnormal inflammatory response, oxidative stress, apoptosis, and proliferation as well as autophagy is accepted as a key intervention for treating RCC in general.

Radix Actinidia chinensis (RAC, Chinese pinyin name Teng Li Gen), also known as kiwi root, is a traditional Chinese medicine (TCM) [12]. RAC has the functions of clearing heat dampness, dispel wind, reducing swelling by detoxification and hemostasis [13]. Studies have shown that RAC contains oleanolic acid, curcumin, emodin, and other components, which can prevent inflammatory response and oxidative stress, suppress cell proliferation, induce apoptosis, and inhibit tumor cell metastasis to attenuate the development of gastric cancer [14], liver cancer [15], lung cancer [16], and so on. However, the specific function and detailed mechanism of RAC in RCC are poorly understood.

Hence, in this study, we aimed to explore whether RAC can attenuate RCC by modulation of inflammation, oxidative stress, apoptosis, proliferation, and autophagy via the PI3K/AKT/mTOR and MAPK pathway, thus providing a novel strategy for treating RCC.

## 2. Materials and Methods

### 2.1. Network Pharmacologic Analysis

#### 2.1.1. Chemical Ingredients and Target Prediction of RAC for RCC Therapy

For building the database of RAC chemical compounds, the active chemical ingredients of RAC were retrieved from the traditional Chinese medicine information database (TCM-ID, http://119.3.41.228:8000/tcmid/), traditional Chinese medicine systems pharmacology (TCMSP, http://lsp.nwu.edu.cn/tcmsp.php), and herbal ingredients' targets (HIT, http://lifecenter.sgst.cn/hit/) based on the relevant screening standard.

TCM-ID, TCMSP, HIT, and search tool for interactions of chemical (STITCH, http://stitch.embl.de/) databases were used to predict the target of the RAC active ingredients. After that, the gene target of the main active compounds of RAC was further selected by relevant screening standard. Upon searching in the GeneCards database (https://www.genecards.org/) using “renal cell carcinoma” as keywords, all gene targets associated with RCC were obtained. Subsequently, the two datasets of gene targets were intersected to obtain gene targets of RAC for treating RCC.

In order to understand the potential mechanisms of TLG for treating RCC, compound-disease-target network analysis was constructed by Cytoscape software (version 3.7.1).

#### 2.1.2. Gene Ontology and Pathway Enrichment Analysis

Gene Ontology (GO) enrichment analysis for overlapping genes was conducted using DAVID web server (https://david.ncifcrf.gov/) to analyze molecular function (MF) and biological process (BP) as well as cellular component (CC) terms. The KOBAS database (http://kobas.cbi.pku.edu.cn/) was adopted for Kyoto Encyclopedia of Genes and Genomes (KEGG) pathway enrichment analyses with setting “Homo sapiens” to find pathways related to the shared targets systematically.

### 2.2. Preparation of RAC Extract

Firstly, weigh 1000 g of RAC, dissolve it in 5000 mL of ethanol, and extract for 3 times by refluxing for 10 h. The extracting solution was combined, evaporated, and dried; then, chromatography separated by silica gel column and gradient elution by dichloromethane, methanol, and acetic acid solutions was performed. The solvent was removed at 400°C, and the powder was collected and dried under vacuum for 24 h. Next, distilled water (100 mL) was added to the 100 g dry product prepared in the above steps; the stock solution was diluted to 50 mg/L, 100 mg/L, and 200 mg/L drug concentrations. The obtained solution was filtered with a 0.22 *μ*m microporous membrane, partitioned, and stored in a 4°C refrigerator for standby use.

### 2.3. Cell Culture

Human renal cancer cell line A498 was grown within RPMI 1640 medium that was supplemented with FBS (10%), penicillin (100 U/mL), and streptomycin (100 U/mL). Then, the A498 cells were cultured under the conditions of 37°C, saturated humidity, and 5% CO_2_. The A498 cells were digested and passaged with trypsin (0.25%).

### 2.4. Animals

Male BALB/c nude mice were supplied by Shanghai SLAC Laboratory Animal Co., Ltd. (China). The mice were reared in a lighting-, humidity-, and temperature-maintained (12 h : 12 h; 50-60%; 20-22°C) animal room with free access to food and drinking water. All animal experiments were performed with the approval of the Animal Ethics Committee of Hangzhou Eyoung Biomedical Research and Development Center (Permit Number: SYXK 2021-0033), and the experiments were conducted according to the guidelines of the Chinese Council on Animal Care.

### 2.5. Establishment of Mouse Model

The mice were randomly and equally divided into control, model, RAC low-dose (RAC-L), RAC middle-dose (RAC-M), and RAC high-dose (RAC-H) groups (*n* = 6/group). Subsequently, the A498 cells (1 × 10^7^/mL, 200 *μ*L) in the logarithmic phase were subcutaneously injected into the right armpit of the mice. The mice in the control group received an equal volume of saline instead. After 1 week of inoculation, the mice in the RAC-L/M/H were administered with RAC at 5, 10, and 20 mg/kg/d, respectively. The mice in the control and model groups were given the same volume of distilled water. RAC and distilled water were given daily for consecutive 24 days via gavage. Beginning on the 7th day, the tumor volume was recorded every 3 days by the formula of 0.5 × width2 × length. The experiment lasted 31 days, the mice were euthanized at the end of the experiment, their blood was collected, and their tumors were resected, weighted, and stored for the following experiments.

### 2.6. Immunohistochemical Staining

The protein expressions of Ki67, VEGF, and PCNA in tumor tissues were measured immunohistochemically. Specifically, the processed sections were covered with citrate buffer for antigen retrieval and incubated with 5% BSA to block unspecific binding. Subsequently, the primary antibodies against Ki67 (1 : 200, ab16667), VEGF (1 : 500, 19003-1-AP), and PCNA (1 : 200, 110205-2-AP) were added to the sections for coincubation at 4°C overnight. Afterwards, the sections were incubated with HRP-conjugated secondary antibodies. Next, the sections were visualized with diaminobenzidine reagent. Upon counterstaining with hematoxylin, the sections were examined by microscopy. The software of ImageJ was employed to quantify the Ki67-, VEGF-, and PCNA-positive cells. The primary antibody against Ki67 were purchased from Abcam, and the other antibodies were purchased from Proteintech.

### 2.7. Histopathology

The hematoxylin and eosin (HE) staining was conducted to assess the pathological changes of tumor tissues. Simply speaking, tumor tissues were fixed with 4% paraformaldehyde, dehydrated using gradient ethanol, and then vitrified with xylene. Next, the tumor tissues were embedded in paraffin, sliced into 5 *μ*m thick serial sections. Subsequently, the sections were dewaxed by xylene and rehydrated with 100 to 75% ethanol. After that, the sections were stained with HE. Subsequently, the slides were sealed and analyzed by a light microscope.

### 2.8. Enzyme-Linked Immunosorbent Assay (ELISA)

ELISA was applied to measure the contents of tumor inflammatory cytokines (IL-2, IL-6, IL-10, and TNF-*α*) and serum oxidative stress factors (SOD, MDA, ROS, and GSH-Px). Specifically, the tumor tissues were homogenized and the homogenates collected to detect the contents of IL-2 (MM-0701 M2), IL-6 (ml002293), IL-10 (ml002285), and TNF-*α* (MM-0132 M1) by ELISA kits. On the other hand, the blood was centrifuged and the serum obtained to measure SOD (ml643059), MDA (LS-F4236), ROS (ml009876-1), and GSH-Px (ml058194) contents by ELISA kits. IL-2 and TNF-*α* ELISA kits were purchased from Meimian, MDA ELISA kit was purchased from LifeSpan BioSciences, and other ELISA kits were purchased from Shanghai Enzyme-linked Biotechnology Co.

### 2.9. Terminal Deoxynucleotidyl Transferase dUTP Nick End Labeling (TUNEL) Assays

TUNEL assays were conducted to assess the apoptosis of the tumor tissues. Firstly, tumor tissues were sliced into 5 *μ*m thick sections followed by fixation, dehydration, transparency, and paraffin embedding. Next, the tissues were dewaxed with xylene, rehydrated by gradient ethanol, followed by permeabilizing with proteinase K solution (Servicebio, G1205). After washing with PBS, the slices were incubated with TUNEL reaction buffer for 1 h in the dark and then observed by fluorescence microscopy.

### 2.10. Western Blot Assay

The protein expressions in tumor tissues were evaluated by western blot. In short, the tumor tissues were treated with RIPA lysate to isolate the total protein. Subsequently, the protein concentration was determined by BCA assay. Next, the same amounts of proteins were separated on 10% SDS-PAGE and transferred to PVDF membranes. After blocking 2 h with 5% skimmed milk, the membranes were incubated with the following primary antibodies overnight for 12 h at 4°C: Bcl-2 (AF6139), Bax (AF0120), Cleaved-caspase 3 (AF7022), Pro-Caspase 3 (Ab32150), Cyclin D1 (DF6386), *β*-actin (AF7018), LC3 (AF5402), Beclin 1 (AF5128), P62 (AF5384), GAPDH (AF7021), p-PI3K (AF3241), PI3K (AF6241), p-AKT (AF0016), AKT (AF6264), p-mTOR (AF3308), mTOR (AF6308), p-P38 (AF3455), P38 (AF6456), p-ERK (AF1015), and ERK (4695 s). After rinsing with TBST, the membranes were reincubated with a corresponding HRP-conjugated secondary antibody for 2 h at 37°C. The immunoreactive bands were visualized by ECL reagent. The densitometry analysis of the immunoreactive bands was performed by ImageJ program software. The antibody against Pro-Caspase 3 was purchased from Abcam, the antibody against ERK was purchased from CST, and the other antibodies were purchased from Affinity. All the primary antibodies were used at a dilution multiple of 1 : 1000, except for *β*-actin and GAPDH, which were diluted 1 : 5000.

### 2.11. Statistical Analysis

The data were presented as mean ± SD and analyzed by SPSS 16.0. One-way ANOVA and SNK tests were applied for multigroup comparison. Kruskal-Wallis *H* test was applied, if variances were not equal. *P* < 0.05 was considered a statistically significant difference.

## 3. Results

### 3.1. Active Compounds and Potential Target of RAC Related to RCC

**Figure 1:**
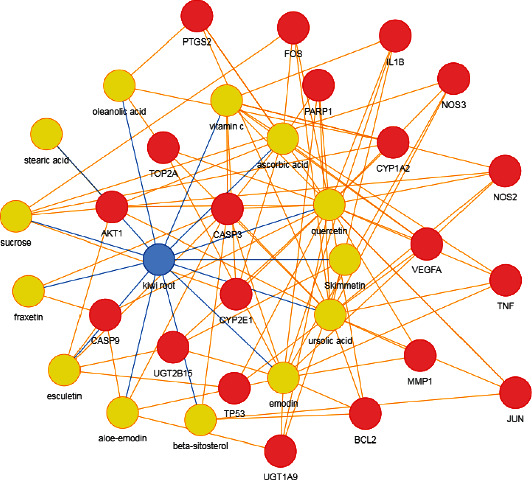
The component-target-disease network of RAC for RCC. Yellow circles represent the compound; red cycles represent the targets. Note: RAC: Radix Actinidia chinensis; RCC: renal cell carcinoma.

After relevant screening, 13 active compounds from RAC were selected, namely, sucrose, stearic acid, oleanolic acid, vitamin C, ascorbic acid, quercetin, skimmetin, ursolic acid, emodin, beta-sitosterol, aloe-emodin, esculetin, and fraxetin. After screening in the databases, 20 potential targets were obtained for these 13 active compounds. The compound-disease-target network of the 13 active ingredients and related 20 targets is exhibited in Figure 1.

### 3.2. Biofunction Analysis

**Figure 2:**
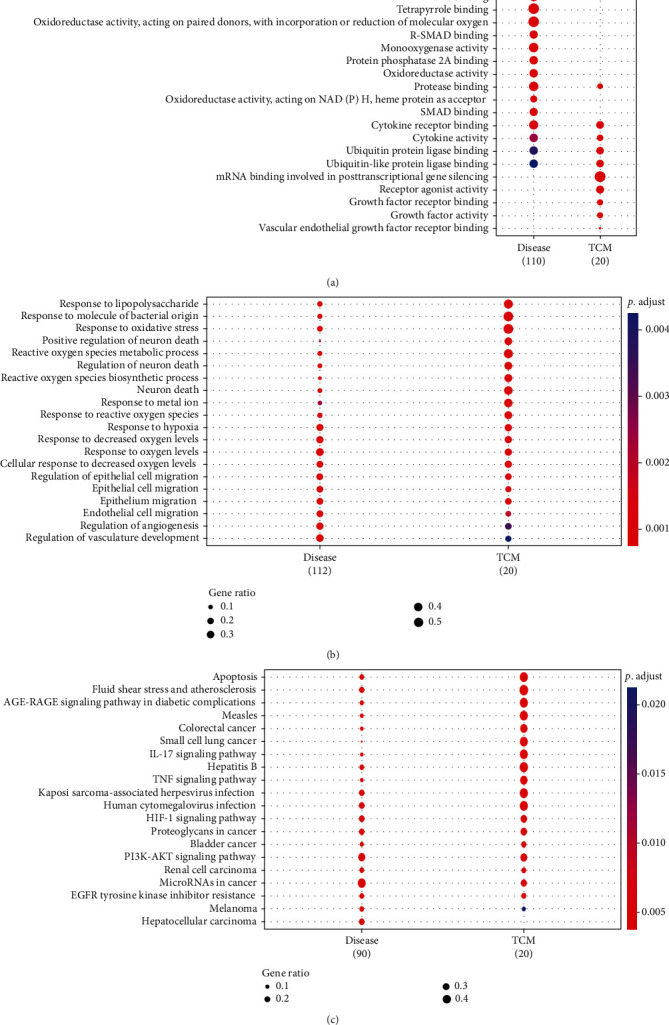
GO analysis and KEGG pathway analysis of the targets: (a) molecular function; (b) biological process; (c) KEGG pathway analysis. Note: GO: Gene Ontology; KEGG: Kyoto Encyclopedia of Genes and Genomes.

Overall, 582 GO terms about the main target of RAC and RCC were identified when adjusted *P* < 0.01, 10 in the MF, 572 in the BP, and 0 in the CC (Figures 2(a) and 2(b)). In the signal pathway enrichment analysis, when *P* < 0.001 was adjusted, there were 35 coassociated pathways between the main target of RAC and RCC (Figure 2(c)), including the RCC-related PI3K/AKT/mTOR and MAPK pathway, and Table S1 displays the top 20 pathways.

### 3.3. RAC Inhibited the Growth of Tumors and Expression of Proliferation Markers in RCC Mice

**Figure 3:**
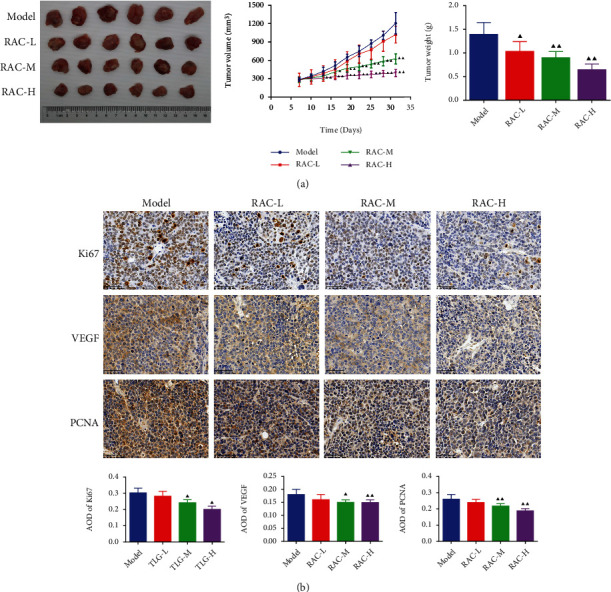
RAC inhibited the growth of tumors in RCC mice. (a) The volume and weight of the tumor. (b) The protein expressions of Ki67, VEGF, and PCNA were measured with immunohistochemistry. ^▲^*P* < 0.05 and ^▲▲^*P* < 0.01*vs.* the model. Results were presented as mean ± SD. *n* = 6 or 3. Note: RAC: Radix Actinidia chinensis; RCC: renal cell carcinoma.

After A498 cells were injected to the nude mice, tumors formed progressively. Then, with the increase of time, the tumor volume of the nude mice in each group increased gradually. As displayed in Figure 3(a), relative to the model group, there was no significant change in the tumor volume in the RAC-L group during the experiment. However, in comparison with the mice in the model group, the tumor volume of the RAC-M/H groups obviously decreased on day 16 and day 13, respectively (*P* < 0.05). Moreover, relative to the model group, the final tumor weight of all-dose group was evidently reduced (*P* < 0.05). Moreover, immunohistochemical staining further confirmed that RAC extract suppressed the expression of the proliferation markers in tumors of RCC mice. As seen in Figure 3(b), obviously weaker Ki67, VEGF, and PCNA staining was observed in the RAC-M/H groups than the model group (*P* < 0.05).

### 3.4. RAC Injured the Tumor Cells in RCC Mice

**Figure 4:**
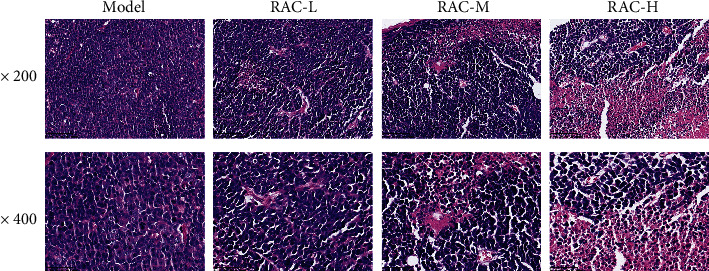
RAC-damaged tumor cell in RCC mice. The pathological changes were evaluated by HE. All the pictures were original magnification × 200 or ×400. *n* = 3. Note: HE: hematoxylin and eosin; RAC: Radix Actinidia chinensis; RCC: renal cell carcinoma.

Figure 4 displayed the images of tumor histopathology in RCC mice. The tumor cells in the model group were overlapped closely packed, and the shape of the tumor cells was irregular. However, after treating with RAC extract for 24 days, the damaged and necrotic tumor tissues were observed.

### 3.5. RAC Prevented the Inflammatory Response and Oxidative Stress in RCC Mice

**Figure 5:**
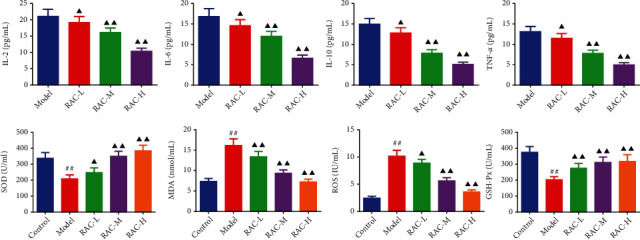
RAC diminished the release of inflammatory cytokines and oxidative stress factors in RCC mice. The contents of tumor IL-2, IL-6, IL-10, and TNF-*α* and serum SOD, MDA, ROS, and GSH-Px in the tumor tissue or serum were measured by ELISA. ^▲^*P* < 0.05 and ^▲▲^*P* < 0.01*vs.* the model. ^#^*P* < 0.05 and ^##^*P* < 0.01*vs.* the control. Results were presented as mean ± SD. *n* = 6. Note: RAC: Radix Actinidia chinensis; RCC: renal cell carcinoma; ELISA: enzyme-linked immunosorbent assay.

As exhibited in Figure 5, the concentrations of IL-2, IL-6, IL-10, and TNF-*α* in the tumor tissue of nude mice in RAC-L/M/H groups were markedly higher than those in the model group (*P* < 0.05). To confirm the antioxidative stress ability of RAC extract, we measured the contents of SOD, MDA, ROS, and GSH-Px in serum of each group. The results of ELISA found that relative to the model group, the contents of SOD and GSH-Px were substantially upregulated while those of MDA and ROS remarkably were downregulated in the RAC-L/M/H groups (*P* < 0.05).

### 3.6. RAC Promoted the Apoptosis of the Tumor Tissue in RCC Mice

**Figure 6:**
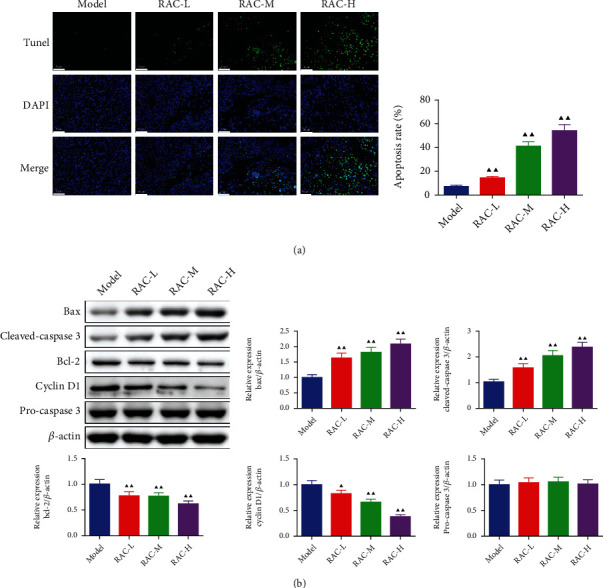
RAC induced the apoptosis of the tumor tissue in RCC mice. (a) The apoptosis was measured by TUNEL. (b) The protein expression of Bax, Cleaved-caspase 3, Bcl-2, and Cyclin D1 was detected by western blot. ^▲^*P* < 0.05 and ^▲▲^*P* < 0.01*vs.* the model. Results were presented as mean ± SD. *n* = 3. Note: RAC: Radix Actinidia chinensis; RCC: renal cell carcinoma; TUNEL: terminal deoxynucleotidyl transferase dUTP nick end labeling.

Apoptosis was scarcely seen in the tumor tissue of the model group; however, as expected, the apoptosis was notably enhanced after treating with RAC (*P* < 0.01, Figure 6(a)). The results were further confirmed by western blot assay, as displayed in Figure 6(b); the expressions of proapoptosis factors (Bax and cleaved-caspase 3) were notably elevated (*P* < 0.01), whereas the expressions of antiapoptotic factor (Bcl-2) and cell cycle regulator (cyclin D1) were markedly blunted after treating with RAC extract (*P* < 0.05).

### 3.7. RAC Induced the Autophagy of the Tumor Cell in RCC Mice

**Figure 7:**
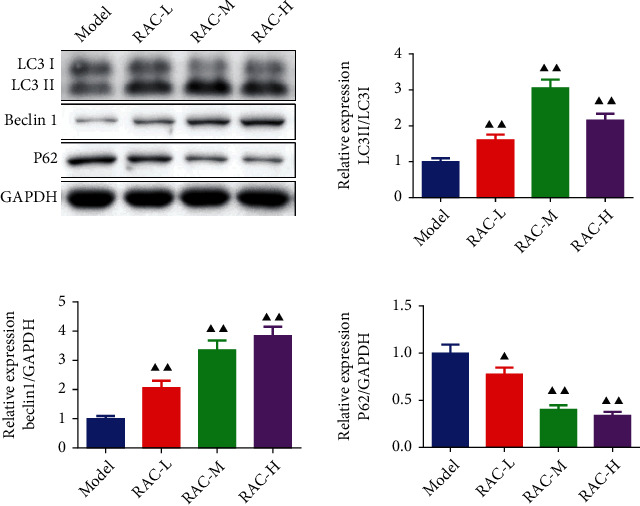
RAC induced the autophagy of the tumor cell in RCC mice. The protein expression of LC3, Beclin 1, and P62 was measured by western blot. ^▲^*P* < 0.05 and ^▲▲^*P* < 0.01*vs.* the model. Results were presented as mean ± SD. *n* = 3. Note: RAC: Radix Actinidia chinensis; RCC: renal cell carcinoma.

The effect of RAC on the expression of autophagy-related protein expression was also detected. As shown in Figure 7, RAC extract notably enhanced the expressions of LC3 I, LC3 II, and Beclin 1 protein and blunted the expressions of P62 protein no matter in the low, middle, or high dose (*P* < 0.05).

### 3.8. RAC Prevented the Activation of the PI3K/AKT/mTOR and MAPK Pathway

**Figure 8:**
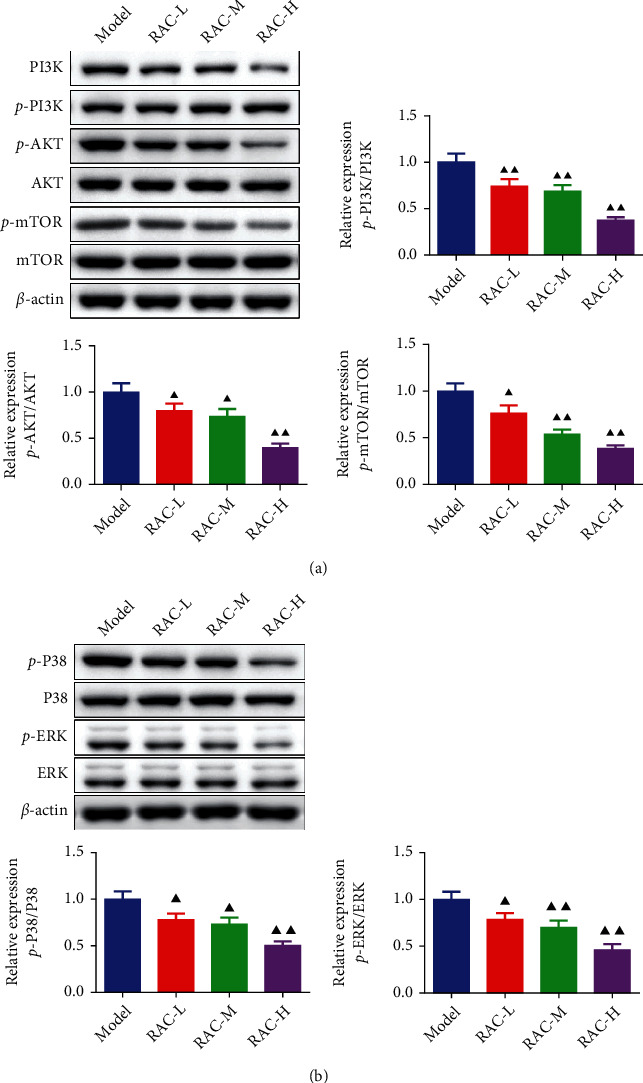
RAC prevented the activation of the PI3K/AKT/mTOR and MAPK pathway. The phosphorylation of PI3K, AKT, and mTOR (a) and P38 and ERK (b) was measured by western blot. ^▲^*P* < 0.05 and ^▲▲^*P* < 0.01*vs.* the model. Results were presented as mean ± SD. *n* = 3. Note: RAC: Radix Actinidia chinensis; RCC: renal cell carcinoma.

As showcased in Figure 8, both RAC-L and RAC-M as well as RAC-H groups notably decreased the phosphorylation of PI3K, Akt, mTOR, P38, and ERK in tumor tissues (*P* < 0.05). Meanwhile, the phosphorylation of PI3K, Akt, mTOR, P38, and ERK in the RAC-H group was lower than that in the RAC-L and RAC-M groups, which indicated that the inhibitory effect of RAC on the PI3K/AKT/mTOR and MAPK pathway may be dose dependent.

## 4. Discussion

RCC represents a prevalent renal parenchymal malignancy that is strongly linked to metabolism [17]. TCM has been confirmed to have managed RCC effectively in many studies [18]. RAC is a commonly used anticancer TCM, which has attracted much attention for the effect of anticancer since its identification [19]. It was reported that some components of RAC, such as ascorbic acid [20], emodin [21], and quercetin [22], could suppress the development and progression of RCC. In this study, we found 13 active ingredients and 20 potential targets of RAC for treating RCC. Meanwhile, the decreased tumor volume, weight, and protein expression of Ki67, VEGF, and PCNA as well as the increased extent of tumor cellular necrosis and damage were observed in RCC mice after treating with middle or high dose of RAC extract. These results indicated that RAC extract could attenuate the development of RCC.

The cause of RCC is still unclear, but inflammation and oxidative stress are contributing to its progression [23]. Soyupek et al. have revealed that the tumor grade of RCC is positively correlated with inflammation score of intratumor and extratumor [24]. Moreover, a published research revealed that oxidative stress would induce the malignant transformation of kidney cells [25]. Consistent with previous studies, our study measured the expressions of inflammatory cytokines (IL-2, IL-6, IL-10, and TNF-*α*) and oxidative stress factors (SOD, MDA, ROS, and GSH-Px) and observed that the inflammatory response and oxidative stress were prevented upon treating with RAC extract. These results suggested that RAC may attenuate RCC via inhibiting inflammation response and oxidative stress.

The PI3K/AKT/mTOR and MAPK pathway is renowned for its activity in the development of RCC [26]. Recently, studies have revealed that the release of inflammatory cytokines, such as IL-2, IL-6, IL-10, and TNF-*α*, and the activity of ROS which initiated RCC are modulated by the MAPK pathway [27]. Meanwhile, numerous studies have found some targets for treating RCC due to their regulatory effect on the PI3K/AKT/mTOR pathway [28]. In the present study, through network pharmacology, we selected the top 20 pathways of RAC involved in treating RCC, including the PI3K/AKT/mTOR and MAPK pathway. Then, in the RCC mouse model, RAC extract downregulated the phosphorylation of PI3K, Akt, mTOR, P38, and ERK, which further demonstrated that RAC might exhibit anti-RCC effect by suppressing the PI3K/AKT/mTOR and MAPK pathway.

Induction of cancer cell necrosis, apoptosis, and autophagy are the three main mechanisms of tumor suppression, and necrosis had been discussed above [29]. A previous study has revealed that G protein alpha inhibitory subunit 1 (G*α*i1) may act as a target for treating RCC, due to inhibition of G*α*i1 which could promote cell apoptosis by downregulating AKT/mTOR and ERK/MAPK pathways [30]. In addition, numerous studies have found that the main components of RAC, such as aloe-emodin [31], emodin [32], and oleanolic acid [33], can induce apoptosis in cancer cells via regulating the MAPK pathway. In the present study, the results of TUNEL staining exhibited that RAC treatment promoted apoptosis in tumor tissue. The alteration in the protein expression of apoptosis-related factors (Bax, Bcl-2, and cleaved-caspase 3) and cell cycle regulator (cyclin D1) presented powerful evidence that RAC extract promoted apoptosis in RCC.

Autophagy, a conserved intracellular process, takes part in the pathogenesis of many diseases, including RCC [34]. Some studies have demonstrated that autophagy can suppress the development of tumor [35]. LC3 and P62 are responsible for maintaining the balance of the formation and degradation of autophagosome [36]. Moreover, Beclin 1 is able to bind to Bcl-2 and then forms Beclin 1/Bcl-2 complex to involve the modulation of autophagy, and the enhanced Beclin 1 expression is related to the elevated autophagosome number [37]. A published research conducted by Guo et al. have reported that quercetin, one of the active ingredients of RAC, can decline the P62 expression and elevate the Beclin-1 and LC3 expression to enhance autophagic response in lung cancer [38] and acute hepatitis [39]. Similar results were found in the treatment of liver cancer with oleanolic acid [40]. In this study, we observed that the expressions of LC3 and Beclin 1 protein were promoted while the expression of P62 was prevented with RAC treatment. The effect of RAC on the induction of autophagy was further confirmed by the suppressed PI3K/AKT/mTOR pathway, which is known to modulate autophagy negatively [41].

Nevertheless, there were two limitations in our study. One was the lack of a positive control group (i.e., pazopanib). The other was the study only performed in vivo experiments, which lacked corresponding cellular experiments to validate the function and mechanism of RAC on RCC. In the future, we will consider adding a positive control group and conduct cellular experiments to better prove the results.

In summary, this study revealed the function and detailed mechanism of RAC in RCC therapy. Specifically, RAC exhibited obvious inhibitory effects on inflammatory response, oxidative stress, proliferation, and PI3K/AKT/mTOR and MAPK pathway and promotion effects on apoptosis and autophagy. All of these revealed that RAC may serve as a novel agent for treating RCC, and PI3K/AKT/mTOR as well as MAPK pathways may be critical targets for RCC therapy.

## Data Availability

The data used to support the findings of this study are available from the corresponding author upon request.

## Supplementary Materials

Supplementary Materials Table S1: top 20 enriched signaling pathways from KEGG analysis.
