# Supplementary material for: Radix Actinidia chinensis Suppresses Renal Cell Carcinoma Progression: Network Pharmacology Prediction and In Vivo Experimental Validation
Source: Anal Cell Pathol (Amst). 2022 Jul 30;2022:3584445. doi: 10.1155/2022/3584445 (PMC9356879; doi:10.1155/2022/3584445)
Supplement: Supplementary Materials — Table S1: top 20 enriched signaling pathways from KEGG analysis. [file 3584445.f1.doc]

Table S1 Top 20 enriched signaling pathways from KEGG analysis

| ID | Description | | p.adjust | Count |
| --- | --- | --- | --- | --- |
| hsa04210 | Apoptosis | | 1.33E-09 | 9 |
| hsa05418 | Fluid shear stress and atherosclerosis | | 1.33E-09 | 9 |
| hsa04933 | AGE-RAGE signaling pathway in diabetic complications | | 2.81E-09 | 8 |
| hsa05162 | Measles | | 2.85E-08 | 8 |
| hsa05210 | Colorectal cancer | | 3.03E-08 | 7 |
| hsa04657 | IL-17 signaling pathway | | 4.08E-08 | 7 |
| hsa04151 | PI3K-Akt signaling pathway | | 0.000491 | 6 |
| hsa05211 | Renal cell carcinoma | | 0.000623 | 3 |
| hsa05222 | Small cell lung cancer | | 4.08E-08 | 7 |
| hsa05161 | Hepatitis B | | 5.13E-08 | 8 |
| hsa04668 | TNF signaling pathway | | 1.09E-07 | 7 |
| hsa05167 | Kaposi sarcoma-associated herpesvirus infection | | 1.39E-07 | 8 |
| hsa01524 | Platinum drug resistance | | 2.32E-07 | 6 |
| hsa04926 | Relaxin signaling pathway | | 2.32E-07 | 7 |
| hsa05163 | Human cytomegalovirus infection | | 3.66E-07 | 8 |
| hsa05142 | Chagas disease (American trypanosomiasis) | | 1.24E-06 | 6 |
| hsa05152 | Tuberculosis | | 1.48E-06 | 7 |
| hsa04010 | MAPK signaling pathway | | 2.03E-06 | 8 |
| hsa05169 | Epstein-Barr virus infection | | 2.46E-06 | 7 |
| hsa05170 | Human immunodeficiency virus 1 infection | | 3.39E-06 | 7 |
|  | |  | | |

Note：KEGG, Kyoto Encyclopedia of Genes and Genomes
